# Advantages of caudal block over intrarectal local anesthesia plus periprostatic nerve block for transrectal ultrasound guided prostate biopsy

**DOI:** 10.12669/pjms.324.9823

**Published:** 2016

**Authors:** Na Wang, Yaowen Fu, Haichun Ma, Jinguo Wang, Yang Gao

**Affiliations:** 1 Na Wang, Department of Anesthesiology, The First Hospital of Jilin University, Jilin, China; 2 Yaowen Fu, Department of Urology, The First Hospital of Jilin University, Jilin, China; 3 Haichun Ma, Department of Anesthesiology, The First Hospital of Jilin University, Jilin, China; 4 Jinguo Wang, Department of Urology, The First Hospital of Jilin University, Jilin, China; 5 Yang Gao, Department of Anesthesiology, The First Hospital of Jilin University, Jilin, China

**Keywords:** Biopsy, Caudal block, Periprostatic nerve block, Prostate, Ultrasound, Transrectal

## Abstract

**Objective:**

To compare caudal block with intrarectal local anesthesia plus periprostatic nerve block for transrectal ultrasound guided prostate biopsy.

**Methods:**

One hundred and ninety patients scheduled for transrectal ultrasound guided prostate biopsy were randomized equally into Group-A who received caudal block (20 ml 1.2% lidocaine) and Group-B who received intrarectal local anesthesia (0.3% oxybuprocaine cream) plus periprostatic nerve block (10 ml 1% lidocaine plus 0.5% ropivacaine) before biopsy. During and after the procedure, the patients rated the level of pain/discomfort at various time points. Complications during the whole study period and the patient overall satisfaction were also evaluated.

**Results:**

More pain and discomfort was detected during periprostatic nerve block than during caudal block. Pain and discomfort was significantly lower during prostate biopsy and during the manipulation of the probe in the rectum in Group-A than in Group-B. No significant differences were detected in the pain intensity after biopsy and side effects between the two groups.

**Conclusions:**

Caudal block provides better anesthesia than periprostatic nerve block plus intrarectal local anesthesia for TRUS guided prostate biopsy without an increase of side effects.

## INTRODUCTION

Transrectal ultrasound (TRUS) guided biopsy is the standard procedure to diagnose prostate cancer.^1^ Different methods of anesthesia have been used to make TRUS guided prostate biopsy a comfortable procedure.^2^ The combination of intra rectal local anesthesia (IRLA) and periprostatic nerve block (PNB) is widely used and is considered to be the preferred method to decrease pain associated with prostatebiopsy.^3^-^5^ However, pain associated with prostate biopsy has double origins, including needle puncture and the manipulation of TRUS probe in anal canal and rectum. IRLA cannot completely eliminate pain associated with TRUS Probemanipulation, besides two punctures for PNB also produce pain.^2^-^5^ Caudal block is an established anesthesia method for urological procedures such as cystoscopy, circumcision as well as TRUS guided prostate biopsy, especially in Japan.^2^ To the best of our knowledge, few studies have reported about advantages of caudal block over IRLA plus PNB for TRUS guided prostate biopsy. We conducted this prospective and randomized study to compare caudal block with IRLA plus PNB for TRUS guided prostate biopsy.

## METHODS

After approval of the institutional ethical committee, this prospective and randomized clinical trial was conducted in our hospital. Study inclusion criteria were prostate specific antigen 4 μg/L or greater, abnormal digital rectal examination and/or TRUS suspicious lesions. Exclusion criteria were American Society of Anesthesiologists (ASA) physical status more than three, previous prostate biopsies, chronic prostatitis, inflammatory bowel disease, anorectal fissure/fistula, active urinary tract infection, bleeding disorder and allergy to local anesthetics.

Informed consent was obtained from 190 patients. They were randomized by a computer-generated schedule and equally assigned to two groups, including Group-A which received caudal block and Group-B which received IRLA plus PNB before TRUS guided prostate biopsy. Anticoagulation or antiaggregant therapy was discontinued seven days before biopsy. As antibiotic prophylaxis, intravenous 4.0 gsulbenicillin sodium or 0.2 g levofloxacin (only when the patient was allergic to sulbenicillin) was administrated one hour before the procedure. A cleaning enema was administrated on the morning of biopsy. All patients were instructed about how to assess the pain and discomfort level using visual analogue scale (VAS) before the procedure.

Patients were placed in left side-lying position during anesthetic block and biopsy. In Group-A, a 5ml syringe with a 0.7-mm, 32-mm needle was used to apply 2ml 0.5% lidocaine at the puncture site for local anesthesia, and then for caudal puncture. After successful puncture, 20 ml 1.2% lidocaine (lidocaine, Shanghai Zhaohui Pharmaceutical CO. LTD, Shanghai, China) was injected for caudal block. The syringe was aspirated before caudal injection to prevent inadvertent injection of lidocaine into blood vessels or subarachnoid space. Caudal block was performed by the same anesthesiologist in this study. If three attempts of caudal puncture failed, caudal block was abandoned and the patient was excluded from the study. Cold tests were used to assess the effect of caudal block. Five minutes after caudal injection, preliminary digital rectal examination was done before insertion of the transrectalprobe (PVT-781-BT, Toshiba, Nasu, Japan) with lubrication of ultrasound gel (Bailesi, Tianjin Xinyan medical equipment CO. LTD, Tianjin, China), and then the biopsybegan. After biopsy, motor block and sensory block level were assessed.

In Group-B, during IRLA, 10 ml 0.3% oxybuprocaine gel (oxybuprocaine hydrochloride gel, Shenyang Luzhou Pharmaceutical CO. LTD, Shenyang, China) was applied around the anal ring, in the anus and rectum 10 minutes before digital rectal examination and introduction of the TRUS probe. There after a 22 gauge, 20 cm spinal needle was used to inject 10ml of a mixture of 1% lidocaine and 0.5% ropivacaine (Naropin, AstraZeneca AB, Sodertalje, Sweden). In Group-B and during PNB, 5 ml of the drug solution were injected on each side into the neurovascular bundles at prostate-bladder-seminal vesicle angle under TRUS guidance. During PNB injection, direct intravascular injection should be avoided by aspiration of the spinal needle. Injection was confirmed by separation of tissue planes under TRUS monitoring. Five minutes later, prostate biopsy began. The procedure of biopsy thereafter was performed in a similar manner in all patients.

Prostate biopsies were performed with a BARD Magnum biopsy gun (MG1522, Bard Company, Covington, U.S.) and an 18 gauge, 200mm TSK topcutbiopsy needle (MGN, TSK Soja No. 1 Factory, Tochigi, Japan) by the same operator using a12-core scheme. Before removing the ultrasound probe at the end of the procedure, the prostate was again visualized to search for any signs of hematoma, and then prostate volume was calculated using the formula for a prolate ellipsoid (width ×length × height × 0.52). After biopsy, a piece of iodoform gauze was packed in the rectum to control bleeding and it was removed at the first time when the patient voided. All patients were hospitalized for one day and observed for two hours after biopsy.

Patients rated pain and discomfort perceived based on a 11-point VAS at various time periods, including T1—during caudal block or periprostatic nerve block, T2—during introduction and presence of the probe in the rectum, T3—duringprostate biopsy, T4—30 minutes after biopsy and T5—a day after biopsy. An attending nurse evaluated the pain scales using visual analogue scale (VAS: 0- no pain; 1, 2, 3- mild pain; 4, 5, 6- moderate pain; 7, 8, 9- severe pain; 10- the worst pain the patient had ever experienced). Patients were blinded to the grouping situation. Complications and prostate cancer detection rate were recorded. Patients were asked about complications when they returned for histological findings three days after biopsy. The overall patient satisfaction was evaluated using a four-point scale (1: poor; 2: moderate; 3: good and 4: excellent). Complications such as lipothymia/syncope requiring intravenous therapy, allergic reaction, hematuria and rectal bleeding requiring hospitalization, acute urinary retention and fever greater than 38.5 were regarded as severe complications.

All data were analyzed using SPSS 17.0 (SPSS Inc, IL, US). The primary endpoint was the difference of pain intensity evaluated by VAS during prostate biopsy. Aminimum 1-point difference in the 11-point VAS score is generally considered clinically significant. To detect a 1-point difference with 90% power and two-sided 5%significance, a minimum of 90 patients per group was needed in this study. Weenrolled 95 patients per group for possible dropout. Differences in VAS scores were calculated using Mann-Whitney U test. The qualitative data were analyzed with the Fisher’s exact test. Statistical significance was defined as *P*<0.05.

## RESULTS

**Fig.1:**
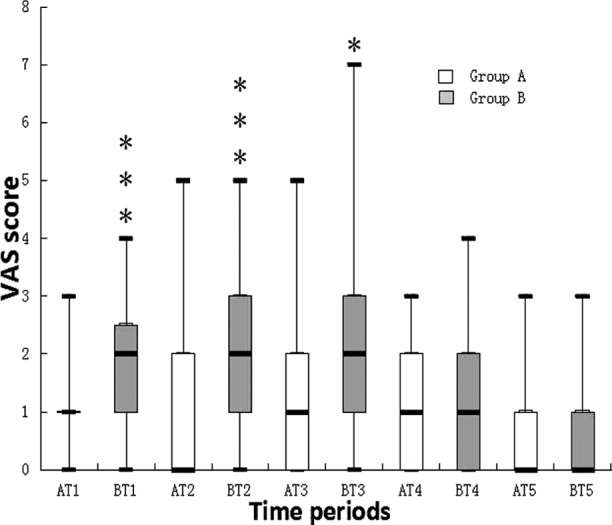
Box plots of VAS scores at various time points. Results are expressed in median. The top and bottom of each box indicate 75th and 25th percentiles and the error bars minimum and maximum values. ***Abbreviations:*** VAS, visual analogue scale; T1, during caudal block or periprostatic nerve block; T2, during introduction and presence of the probe in the rectum; T3, during biopsy procedure; T4, 30 minutes after the procedure and T5, a day after the procedure; Group-A, the caudal block group; Group-B, The intrarectal local anesthesia and periprostatic nerve block group. * indicates *P*<0.05, *** indicates *P*<0.001.

The two groups did not differ in age, serum prostate specific antigen, total prostate volume and cancer detection rate. (Table-I) VAS scores were lower during T1, T2 and T3 in Group-A than in Group-B. No significant differences were noted in VAS scores during T4 and T5 between the two groups. (Table-II & Fig.1) The failure rate of caudal puncture was three out of 95. Patients with successful caudal puncture had positive cold test. Two patients in Group-A felt numb in their feet soles immediately after prostate biopsy. No motor blockade was observed and all patients could walk without assistance right away after the biopsy.

**Table-I:** Patients’ characteristics.

	Group-A (n=92)	Group-B (n=95)	P-value
Age (year)	68.4±6.5	67.8±7.1	0.547
Weight (kg)	59.7±7.3	61.0±7.9	0.244
ASA = 1 \* ROMAN I/ = 2 \* ROMAN II (n)	81/11	78/17	0.308
Prostate specific antigen (μg/L)	29.5±26.6	25.7±22.5	0.292
Prostate volume (ml)	57.9±16.3	62.4±23.3	0.128
Biopsy duration (second)	265.2±46.2	273.4±51.7	0.254
Cancer detection rate (%)	33.7% (31/92)	30.5% (29/95)	0.754

Data are presented as mean±standard deviation or number of patients. ***Abbreviations:*** n, number of patients; ASA, American Society of Anesthesiologists; Group-A, the caudal block group; Group-B, the transrectal local anesthesia and periprostatic nerve block group.

**Table-II:** VAS scores at various time points.

VAS	Group-A (n=92)	Group-B (n=95)	P-value
T1	1.1±0.6	1.7±1.1	<0.001
T2	0.9±1.4	1.8±1.4	<0.001
T3	1.4±1.3	1.9±1.6	0.010
T4	0.9±1.1	1.1±1.1	0.27
T5	0.4±0.7	0.5±0.9	0.44

Data are presented as mean±standard deviation. ***Abbreviations:*** T1—caudal block or periprostatic nerve block, T2—during introduction and presence of the probe in the rectum, T3—biopsy procedure, T4—30 minutes after the procedure and T5—a day after the procedure; n, number of patients; Group-A, the caudal block group; Group-B, the transrectal local anesthesia and periprostatic nerve block group.

No differences were found in side effects associated with anesthesia and biopsy between the two groups. Thirteen patients with prostatic hypertrophy were catheterized due to urinary retention after biopsy, 9 in Group-A, 4 in Group-B. (Table-III). The patients in Group-A were more satisfied than those in Group-B. (Table-IV).

**Table-III:** Side effects.

	Group-A (n=92)	Group-B (n=95)	P-value
Rectal massive bleeding	0	0	1.000
Prolonged macroscopic hematuria	1	3	0.621
High fever	2	4	0.682
Epididiymitis	0	0	1.000
Urinary retention	6	4	0.532
Vasovagal syncope	0	0	1.000
Total Number	9	11	0.814

Data are presented asnumber of patients. ***Abbreviations:*** n, number of patients; Group-A, the caudal block group; Group-B, the transrectal local anesthesia and periprostatic nerve block group.

**Table-IV:** Patient overall satisfaction.

	Poor	Moderate	Good	Excellent
Group-A (n=92)	3 (3.3%)	11 (12.0%)	35 (38.0%)	43 (46.7%)
Group-B (n=95)	5 (5.3%)	18 (18.9%)	49 (51.6%)	23 (24.2%)

*P*=0.014, data are presented as number of patients (%). Abbreviations: n, number of patients; Group-A, the caudal block group; Group-B, the transrectal local anesthesia and periprostatic nerve block group.

## DISCUSSION

TRUS guided prostate biopsy is associated with obvious pain in approximately a fourth of patients.^6^,^7^ The combination of IRLA and PNB is widely used for pain control during the procedure, but the performance of IRLA and PNB delivers pain and discomfort.^2^-^4^ The current study suggests that caudal block effectively decreases pain and discomfort during TRUS guided prostate biopsy compared with IRLA and PNB, and what’s more, caudal block can reduce and even completely eliminate pain and the discomfort related to TRUS probe manipulation which may be more painful than biopsy.^8^ As an additional advantage in contrast to PNB, caudal block provides perianal analgesia and anal sphincter relaxation, making it much easier to maneuver the TRUS probe even in painful anal conditions (such as anal fissure orhaemorrhoids). The caudal block can block sacrococcygeal nerves which in nervatethe whole perineum involving the perianal region, rectum and prostate gland, so it can consequently decrease pain related to TRUS probe introduction and biopsy.^9^-^11^

The result of the present study is consistent with those reported by Ikuerowo and Cesur.^10^,^11^ They reported that caudal block could significantly decrease the level of pain, but not compared with PNB. However, Horinaga et al. reported that the caudal block with 10 ml 1% lidocaine did not provide as effective anesthesia and post procedural analgesia as IRLA plus PNB with the same dose of lidocaine for prostate biopsy.^12^ The different findings in the present study and in the study by Horinaga et al. may result from the smaller volume and lower concentration of lidocaine for caudal block in the latter. Thus, theoretically, an appropriate caudal anesthetic agent with proper volume and concentration might be the optimal method for prostate biopsy. In our study, all patients who were given caudal injection had positive cold test results and received effective caudal anesthesia. Ikuerowo et al. reported that the rate of ineffective analgesia was as high as 17.7% when caudal block was applied.^10^ Their dose was written as 2mg/kg of plain xylocaine, but no indication of the concentration and volume was displayed, so it is hard to tell the reason about the high ineffective rate. There are 3 out of 95 patients excluded from the present study for failure of caudal puncture. The failure rate is not high. Moreover, the application of ultrasound which is kept on hand for prostate biopsy will help with precise localization of the sacral hiatus.^13^ A major concern about caudal block is urinary retention in which no significant difference was found in this study.

Despite its efficacy, caudal block requires presence of an anesthetist, giving the entire procedure a greater cost and reducing possibility of performing prostate biopsy in an outpatient setting. This economic limitation must be weighed against the superior pain control of caudal block.

### Limitations of the study

Our study was not designed to perform such a sub-analysis as specific assessment of VAS scores based on age and prostate volume. However, similar to that reported by others, we perceived that elderly patients and those with a smaller prostate seemed to have lower VAS pain scores.^4^,^14^

## CONCLUSION

Our study indicates that caudal block with 20 ml 1.2% lidocaine provides more effective and comfortable anesthesia than the combination of IRLA and PNB for TRUS guided prostate biopsy.
